# Thoracoscopic esophagectomy with left recurrent laryngeal nerve monitoring for thoracic esophageal cancer in a patient with a right aortic arch: a case report

**DOI:** 10.1186/s40792-020-00819-8

**Published:** 2020-03-30

**Authors:** Yamato Ninomiya, Junya Oguma, Soji Ozawa, Kazuo Koyanagi, Akihito Kazuno, Miho Yamamoto, Kentaro Yatabe

**Affiliations:** 1 Department of Gastroenterological Surgery, Tokai University School of Medicine, 143 Shimokasuya, Isehara, Kanagawa 259-1193 Japan; 2 Department of Esophageal Surgery, National Cancer Center Hospital, 5-1-1 Tsukiji, Chuo-ku, Tokyo, 104-0045 Japan

**Keywords:** Esophageal cancer, Right aortic arch, Thoracoscopic esophagectomy, Recurrent laryngeal nerve, Intraoperative nerve monitoring

## Abstract

**Background:**

Surgery for cases of thoracic esophageal cancer with a right aortic arch is rare, and the anatomic abnormalities in such patients necessitate a different surgical approach. Since the position of the recurrent laryngeal nerve often differs from the usual in these cases, the lymph node dissection around the recurrent laryngeal nerve, which is an important step in surgery for thoracic esophageal cancer, requires careful attention. There are some reports on the usefulness of intraoperative recurrent laryngeal nerve monitoring during esophageal cancer surgery. Herein, we report a case of successful thoracoscopic esophagectomy for esophageal cancer in a patient with a right aortic arch using intraoperative recurrent laryngeal nerve monitoring.

**Case presentation:**

A 70-year-old man was diagnosed as having esophageal cancer (Ut, type 0-IIc, T1b/MtLt, type 0-IIc, T1b, N2, M0, cStage II) and was treated by neoadjuvant chemoradiotherapy followed by radical surgery. Preoperative CT examination revealed a right aortic arch, and based on the findings of 3D-CT, we classified the right aortic arch as type IIIB1 (Edwards classification), which is the most frequent type of right aortic arch. We performed thoracoscopic esophagectomy via a left thoracic approach with the patient placed in the prone position, cervical esophagogastric conduit reconstruction via the retrosternal route, and three-field lymph node dissection. Although Kommerell’s diverticulum could be easily confirmed, the descending aorta took a meandering course, making it difficult for the esophagus to be mobilized and detached and therefore also to identify the ductus arteriosus and left recurrent laryngeal nerve. Intraoperative recurrent laryngeal nerve monitoring using NIM-RESPONSE® 3.0 (Medtronic Japan, Tokyo, Japan) allowed the position of the left recurrent laryngeal nerve to be accurately determined, and upper mediastinal lymph node dissection and mobilization of the upper thoracic esophagus were performed safely. Postoperatively, the patient showed no evidence of recurrent laryngeal nerve palsy, but needed conservative treatment for anastomotic leakage. The patient was discharged 46 days after the surgery.

**Conclusion:**

It was suggested that intraoperative recurrent laryngeal nerve monitoring is useful in esophageal cancer with a right aortic arch undergoing surgery, in whom anatomic abnormalities of the recurrent laryngeal nerve can be expected.

## Background

Right aortic arch (RAA), which occurs during the process of aortic development, is a rare congenital vascular malformation that occurs in approximately 0.1% of the adult population [1]. Many patients with RAA also show heterotaxia, although the majority do not show heterotaxia. Three types of aortic arch anomalies have been identified based on the theoretical concept of development of the aortic arch, and RAA belongs to group III of this classification [2, 3]. Surgery for thoracic esophageal cancer in patients with a RAA is rare, and the anatomic abnormalities encountered in such patients necessitate a different surgical approach [4–6]. Since the position of the recurrent laryngeal nerve (RLN) often differs from the usual in these cases, the lymph node dissection around the RLN, which is an important step in surgery for thoracic esophageal cancer, requires careful attention. We performed 3D-CT before the surgery in this patient with thoracic esophageal cancer with a RAA, for three-dimensional confirmation of the abnormalities in the vascular positions. During the surgery, we approached the left thoracic cavity by thoracoscopic surgery with the patient placed in the prone position. Furthermore, we performed mediastinal lymph node dissection and esophagectomy safely, without nerve damage, by confirming the position of the left RLN and monitoring its function during the surgery.

## Case presentation

**Fig. 1:**
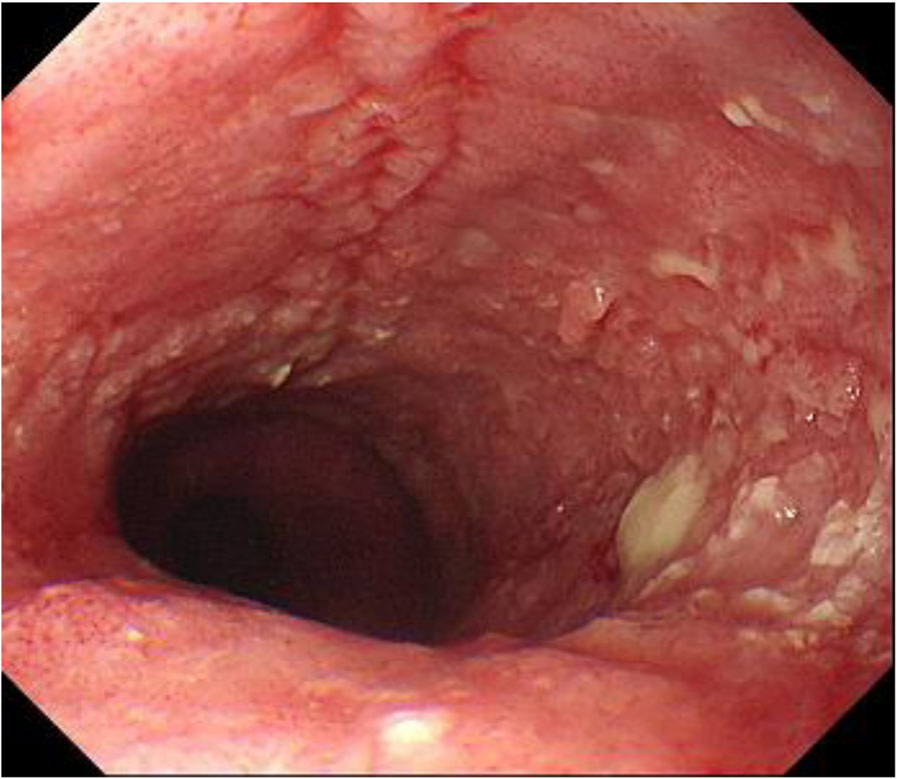
Preoperative upper gastrointestinal endoscopy findings. Endoscopic findings before treatment. A lesion was found in the middle thoracic esophagus, and biopsy revealed the diagnosis of squamous cell carcinoma

A 70-year-old man consulted a neighborhood doctor with a history of discomfort in swallowing and underwent upper gastrointestinal endoscopy. The examination showed mucous membrane irregularities in the thoracic esophagus (Fig. 1), biopsy revealed squamous cell carcinoma, and the patient was referred to our hospital. We made the diagnosis of esophageal cancer (Ut, type 0-IIc, T1b/MtLt, type 0-IIc, T1b, N2, M0, cStage II) and planned to treat the patient by radical surgery after neoadjuvant therapy. The patient was enrolled in a clinical trial of neoadjuvant therapy for esophageal cancer (JCOG1109) and was assigned to the neoadjuvant chemoradiotherapy group (2 courses of CF therapy: cisplatin 75 mg/m^2^, 5-fluorouracil 1000 mg/m^2^, and radiation: 41.4 Gy, 1.8 Gy × 23 fr).

**Fig. 2:**
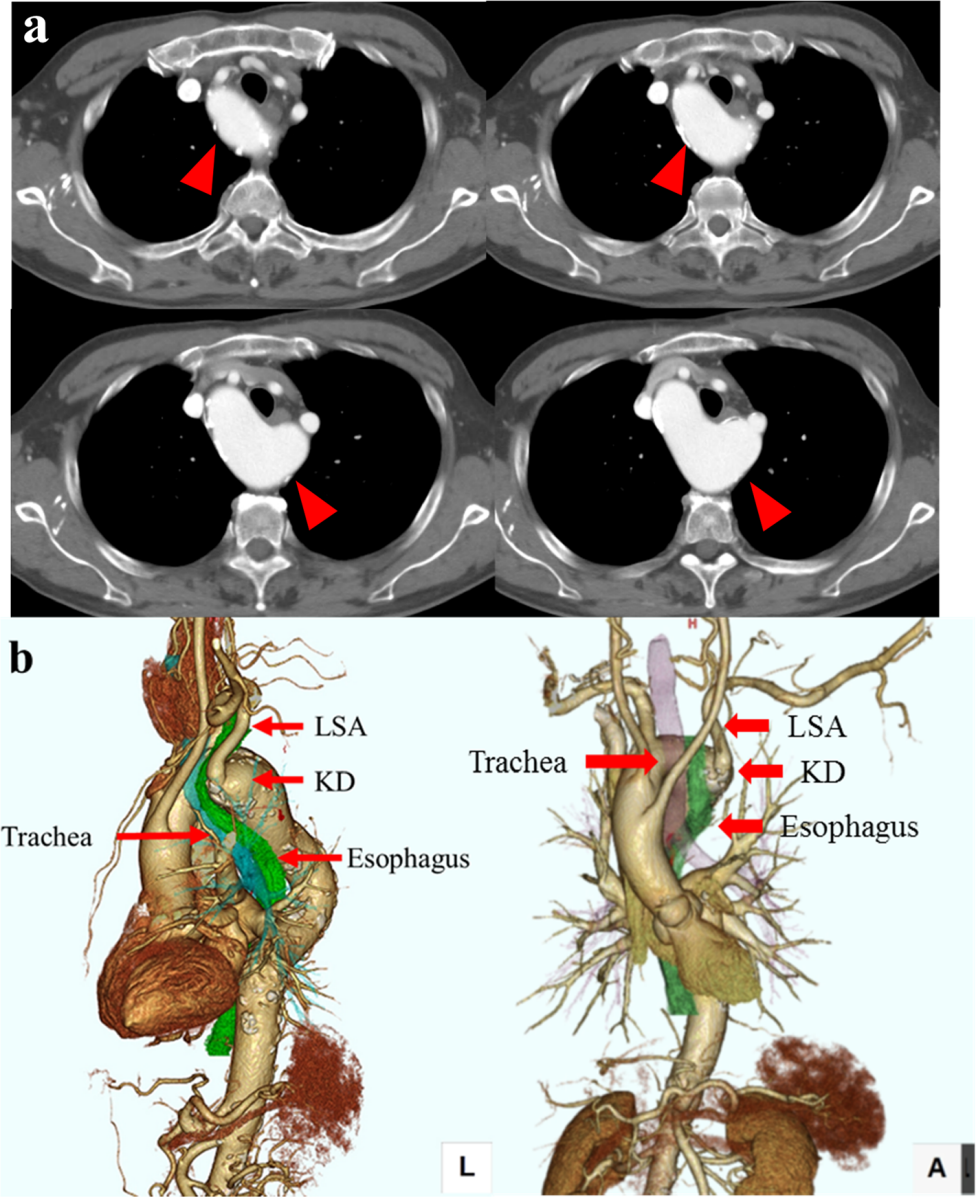
Preoperative CT findings. **a** Transverse image: the right aortic arch (arrow) surrounding the trachea and esophagus from the dorsal side. **b** 3D-CT findings: Edwards classification type IIIB1, with Kommerell’s diverticulum at the origin of the descending aorta, from which the left subclavian artery can be seen to be bifurcating ectopically. LSA: left subclavian artery, KD: Kommerell’s diverticulum

**Fig. 3:**
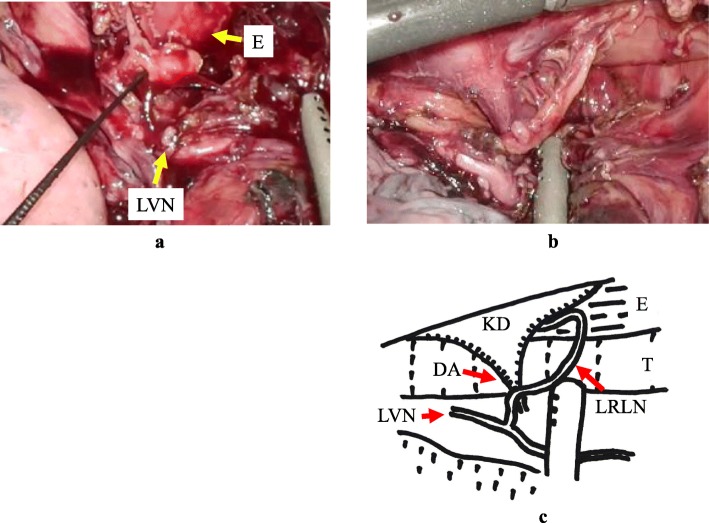
Intraoperative findings. **a** Identification of the left recurrent laryngeal nerve was performed by the needle-type sensor of the nerve integrity monitor. **b** Dissection around the left recurrent laryngeal nerve that encircled the ductus arteriosus has been completed. **c** Schematic illustration. E: esophagus, T: trachea, LVN: left vagus nerve, KD: Kommerell’s diverticulum, DA: ductus arteriosus, LRLN: left recurrent laryngeal nerve

Preoperative CT examination revealed a RAA (Fig. 2a), and based on the findings of 3D-CT, we classified the RAA as type IIIB1 (Edwards classification), which is the most frequently encountered type of RAA (Fig. 2b). We performed thoracoscopic esophagectomy via a left thoracic approach with the patient placed in the prone position, cervical esophagogastric conduit reconstruction via the retrosternal route, and three-field lymph node dissection. Although Kommerell’s diverticulum could be easily confirmed, the descending aorta took a meandering course, making it difficult for the esophagus to be mobilized and detached and therefore also to identify the ductus arteriosus (DA) and left RLN. Continuous and intermittent intraoperative RLN monitoring using NIM-RESPONSE® 3.0 (Medtronic Japan, Tokyo, Japan) allowed the position of the left RLN to be accurately determined (Fig. 3a), and upper mediastinal lymph node dissection and mobilization of the upper thoracic esophagus were performed safely (Fig. 3b, c). The right RLN was considered to be recurrent in the aortic arch, but could not be identified during thoracic surgery. Therefore, intraoperative right RLN monitoring was not performed, and lymph nodes along the right RLN were dissected through a neck incision. Postoperatively, the patient showed no evidence of RLN palsy, but needed conservative treatment for anastomotic leakage. The patient was discharged 46 days after the surgery. Histopathological examination revealed complete response of the primary tumor and lymph node metastasis in 101L. A follow-up examination performed 22 months after the operation revealed no evidence of recurrence. Although intraoperative nerve monitoring for esophageal cancer surgery is not covered by public medical insurance, its cost was reimbursed as research expenses, as the procedure was conducted with the approval of the institutional review board of Tokai University Hospital.

## Discussion

**Fig. 4:**
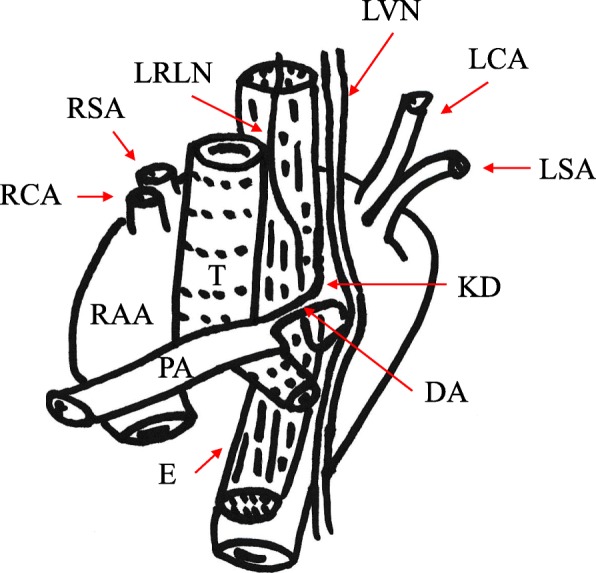
Schematic illustration of the mediastinal structure of the present case. The trachea and esophagus were encircled completely by the right aortic arch. The ductus arteriosus connected Kommerell’s diverticulum to the pulmonary artery. The left recurrent laryngeal nerve passes behind the ductus arteriosus and ascends posteriorly. RAA: right aortic arch, KD: Kommerell’s diverticulum, DA: ductus arteriosus, LRLN: left recurrent laryngeal nerve, LVN: left vagus nerve, LSA: left subclavian artery, LCA: left common carotid artery, RSA: right subclavian artery, RCA: right common carotid artery, PA: pulmonary artery, E: esophagus, T: trachea

The present case had an aberrant left subclavian artery and was classified as having type IIIB1 RAA, which is the most frequently occurring type of RAA, according to the preoperative 3D-CT findings. The aortic diverticulum, known as Kommerell’s diverticulum, was identified at the beginning of the descending aorta, and the DA connected Kommerell’s diverticulum to the pulmonary artery. The left RLN is known to pass behind the DA and ascend posteriorly [7]. Therefore, we thought that it was important to confirm the position of the DA and left RLN during the operation. Intraoperative findings were as expected based on preoperative examination (Fig. 4).

Esophagectomy for esophageal cancer patients with a RAA is rare. Patients with a RAA often have mediastinal vascular malformations, with their anatomic profiles differing from the normal anatomy. Approach of the esophagus via a right thoracic approach is difficult in these cases, because the esophagus is located on the side opposite to the right aortic arch and right descending aorta, and the left approach is most often selected. The disadvantage of the left approach is that it is difficult to identify the right RLN that reverts the aortic arch and to dissect lymph nodes along the right RLN [8]. In this case, preoperative examination showed no evidence of lymph node metastasis around the right RLN, so lymph node dissection was performed through the neck insision alone.

In recent years, thoracoscopic surgery has become popular for esophagectomy in patients with esophageal cancer, and its minimally invasive nature and advantage of offering magnified views have been reported as favorable features [9]. There are reports of three cases in the literature, including this case reported herein, of thoracoscopic esophagectomy with mediastinal lymph node dissection performed for thoracic esophageal cancer in patients with a RAA [10, 11]. Although there were numerous difficulties in the thoracoscopic esophagectomy due to anatomical abnormalities, identification and detachment of the DA, which are particularly important steps in this surgical procedure, could be performed safely, taking advantage of the magnified views offered by thoracoscopy.

In this case, it was important to identify the left RLN and to perform esophagectomy without nerve damage, and we decided to use intraoperative RLN monitoring. Intraoperative nerve monitoring is commonly performed in the field of otolaryngologic and head and neck surgery, and numerous reports of its beneficial effect in preventing RLN palsy have been published [12, 13]. In this case, it was particularly difficult to grasp the anatomy around the DA, but the position of the left RLN could be easily confirmed by the magnified views offered by the thoracoscopic approach and RLN monitoring, and esophagectomy was successfully performed without nerve damage. To the best of our knowledge, this the first case report of thoracoscopic esophagectomy performed with intraoperative RLN monitoring in an esophageal cancer patient with a RAA.

Recently, there have also been other occasional reports of intraoperative monitoring of the RLN during esophageal cancer surgery [14, 15]. The significance of performing intraoperative nerve monitoring routinely for the purpose of preventing RLN paralysis needs to be carefully examined further, although intraoperative nerve monitoring certainly appears to be useful in cases with anatomical abnormalities of the RLN, as in this case.

Herein, we report safely performing thoracoscopic esophagectomy after preoperative 3D-CT confirmation of vascular abnormalities and intraoperative RLN monitoring in an esophageal cancer patient with a RAA.

## Conclusion

It was suggested that intraoperative recurrent laryngeal nerve monitoring is useful in esophageal cancer with a right aortic arch undergoing surgery, in whom anatomic abnormalities of the recurrent laryngeal nerve can be expected.

## Data Availability

Data sharing is not applicable to this article, as no datasets were generated or analyzed during the current study.

## Abbreviations

RAA: Right aortic arch
RLN: Recurrent laryngeal nerve
DA: Ductus arteriosus
